# *Clostridium*: Transmission *difficile*?

**DOI:** 10.1371/journal.pmed.1001171

**Published:** 2012-02-07

**Authors:** Stephan Harbarth, Matthew H. Samore

**Affiliations:** 1 Infection Control Program, University of Geneva Hospitals and Medical School, Geneva, Switzerland; 2 Veterans Affairs Salt Lake City Health Care System, Division of Epidemiology, University of Utah School of Medicine, Salt Lake City, Utah, United States of America

## Abstract

Stephan Harbarth and Matthew Samore discuss the implications, and the limitations, of new research that might indicate that most Clostridium difficile cases are imported into hospitals.

**Linked Research Article**

This Perspective discusses the following new study published in *PLoS Medicine*:

Walker AS, Eyre DW, Wyllie DH, Dingle KE, Harding RM, et al. (2012) Characterisation of *Clostridium difficile* Hospital Ward–Based Transmission Using Extensive Epidemiological Data and Molecular Typing. PLoS Med 9(2): e1001172. doi:10.1371/journal.pmed.1001172

A population-based study in Oxfordshire (UK) hospitals by Sarah Walker and colleagues finds that in an endemic setting with good infection control, ward-based contact cannot account for most new cases of *Clostridium difficile* infection.

## *Clostridium difficile* Infection: Difficult to Control?

*Clostridium difficile* can cause large-scale outbreaks of diarrhea [1],[2]. Significant progress has recently been achieved to improve treatment of symptomatic *C. difficile* disease [3]. But hospitals affected by *C. difficile* infection still face challenges in the effort to control endemic *C. difficile* infections, which may be related to overuse of antibiotics (e.g., fluoroquinolones, cephalosporins), problems in cleaning services, and poor isolation practices [4],[5]. Furthermore, current diagnostic tests for *C. difficile* are not sensitive enough [6] and diagnosis can be delayed [7].

Evidence for the rate of nosocomial acquisition of *C. difficile* and the likelihood of within-hospital transmission from patients to patients of *C. difficile* infection remains scarce, so an improved evidence base could help improve infection control strategies [8]. Only a few studies have examined in detail the prevalence of *C. difficile* in hospital patients upon admission and nosocomial transmission rates of *C. difficile* infection [9]. For instance, 15 years ago, Samore et al. reported that for most epidemiologically linked contacts of *C. difficile* cases, positive cultures for *C. difficile* did not result from transmission from the presumed index case [8]. However, this and other studies were conducted before the emergence of new hypervirulent *C. difficile* strains and might not reflect the current epidemiology of *C. difficile* transmission.

## *Clostridium difficile* Infection: Difficult to Transmit?

In a new study published in this issue of *PLoS Medicine*, Sarah Walker and colleagues examine the epidemiology of *C. difficile* infection, focusing on the role of within-hospital transmission among ward patients. The investigators used a simplified model that was populated with observational data from one National Health Service (NHS) Hospital Trust in the United Kingdom, a country that introduced compulsory surveillance with mandatory *C. difficile* testing of all elderly inpatients with diarrhea in 2008 [10]. Surprisingly, based on the results of their network analysis combined with molecular strain typing, up to three-quarters of patients with *C. difficile* infection did not acquire their infecting *C. difficile* strain during their hospital stay. Using time intervals, strain types, and patient location as plausibility checks, the authors propose that within-hospital transmission accounted for a relatively small number of the overall *C. difficile* cases detected. However, the rates of transmission varied in different specialty wards, with renal and transplant wards having the highest documented rates. Most of the cases of *C. difficile* that were attributed to within-hospital transmission occurred shortly after the onset of symptoms of the index case, suggesting that the hospital environment was not, as has previously been claimed, a long-lasting reservoir for this pathogen [7]. Overall, this study suggests that alternative explanations need to be sought for the origin of most of the new onset cases of *C. difficile* infection.

## Moving On—What Do We Need to Know Next about *C. difficile* Transmission?

This impressive study addresses an important question—to what extent can we control *C. difficile* infection by prevention of transmission from symptomatic *C. difficile* infection cases in hospitals? However, there are limitations to the approach chosen in this study, including several possible sources of bias already mentioned by the authors (e.g., selection, misclassification, and information biases).

Other potential limitations not considered in this study include the possibility of inter-ward transmission. Patients from different wards might, for instance, be transported to common sectors of the hospital for procedures and diagnostic tests, e.g., X-rays. Potential vectors of transmission, including equipment and health care workers who might care for patients on different wards, could be similarly mobile. In this study, wards were small relative to the hospital size. It is likely that, on average, many more symptomatic *C. difficile* cases were housed on “other” wards than on the “same” ward. Even though rates of *intra*-ward transmission *per infected case* were probably significantly higher than rates of *inter*-ward transmission *per infected case*, the absolute number of inter-ward transmissions may have, in fact, exceeded the number of intra-ward transmissions. Second, the poor sensitivity of the Enzyme Immuno-Assay (EIA) testing method for *C. difficile* diagnosis may have ignored a potentially significant pool of undiagnosed *C. difficile* patients (which could have been selected as controls, introducing misclassification bias). Third, antibiotic exposure data were not recorded, which could have biased the dates of onset of symptoms and cross-transmission. Finally, transmission events linked to asymptomatic carriers were not routinely detected [11].

## Practical Implications

The two key practical questions related to this study are 1) how much benefit is accrued by blocking transmission from symptomatic *C. difficile* infection cases; 2) what proportion of the *C. difficile* infections that are attributed to within-hospital transmission instead represent already-infected individuals who come into the hospital carrying toxigenic *C. difficile* strains in their gut flora. The study by Sarah Walker and colleagues cannot provide definitive answers to these questions because it has significant limitations with respect to both issues. The study cannot answer question 1, about benefit accrued by blocking *C. difficile* transmission, because it did not examine inter-ward transmission. Further, it cannot tell us how many patients came in already colonized or infected because it did not examine asymptomatic *C. difficile* carriage upon admission and discharge. Attempting to interpret the results of this study with respect to these practical issues highlights the need to utilise models that account for the non-linear dynamics of spread of *C. difficile*.

## Future Studies

Further studies are needed to elucidate answers to the two key questions we have identified above. Investigations should examine the possibility of transmission from falsely EIA-negative symptomatic patients, asymptomatic carriers (patients or health care workers), and community acquisition with importation of *C. difficile* into the hospital setting [12], and this might require both more data and the use of more advanced transmission models such as hidden Markov models.

More detailed screening data, such as a study that reported screening of asymptomatic *C. difficile* carriers in a large prospective cohort [13], and new models will help to answer the question of whether *C. difficile* is less of an institutional and more of a community problem than has previously been thought. Proving that the majority of nosocomial *C. difficile* infections are actually imported into hospitals (with toxigenic *C. difficile* strains being already present on admission) would be “*revolutionary*”—however, we believe that the evidence generated by this study, albeit tantalizing, is not yet sufficient to prove this hypothesis.
